# Isolation and characterization of a broad-spectrum *Salmonella* phage targeting featural foodborne serotypes

**DOI:** 10.3389/fmicb.2026.1827076

**Published:** 2026-05-26

**Authors:** Ling Zhang, Chan Zhong, Mei Yang, Mei Shu, Jin-Hua Zhang, Jun Li, Guo-Ping Wu

**Affiliations:** 1 College of Animal Science and Technology, Jiangxi Agricultural University, Nanchang, China; 2 College of Food Science and Engineering, Jiangxi Agricultural University, Nanchang, China

**Keywords:** anti-biofilm, broad-specturm, lettuce, multidrug-resistant *Salmonella*, O antigen

## Abstract

**Background:**

The pathogen-specific lytic activity of bacteriophages has rendered them a highly promising antibacterial agent to control *Salmonella*, a critical foodborne pathogen with increasing antibiotic resistance. However, the issue of phage-resistant strains severely hindered phage applications.

**Methods:**

This study isolated a broad-spectrum bacteriophage JN06 from wastewater. The morphology/host range, infection capacity/stability and genome characteristics of JN06 were determined. Phage JN06 was evaluated in both biofilm inhibition and lettuce decontamination, followed by inoculation with *Salmonella*. The receptor for phage JN06 was identified by comparing adsorption abilities in resistant derivatives as well as mutants of gene deletion with *S. enteritidis* SMT.

**Results:**

JN06 (classified into *Jerseyvirus* based on nucleotide homology) can lyse *Salmonella* strains of various serotypes, with a short latent period (6 min), a large burst size (131 PFU/infected cell), and high stability at different temperatures (4 °C-70 °C) and pH (2–12). Furthermore, no determinants associated with lysogenicity, virulence, or antibiotic resistance were detected in its genome. Notably, it exhibited a biofilm formation inhibition rate of 86.58%, and completely controlled *Salmonella* from artificially contaminated lettuce. O-antigen was identified as the likely adsorption recipient, and molecular docking revealed that the specific binding between its tail fiber protein and the main chain of Gal-initiated O-antigen, D-Man*p*-(1 → 4)-L-Rha*p*-(α1 → 3)-D-Gal*p*, underlying JN06’s lytic activity against multiple serotypes of *Salmonella*.

**Conclusion:**

Collectively, phage JN06 is a highly promising antibacterial agent for controlling Salmonella, benefiting food processing and disease prevention.

## 1 Introduction

*Salmonella* is one of the major causes of foodborne diseases worldwide, posing significant threats to public health and food safety (Luo et al., 2023). It is responsible for substantial morbidity and mortality, particularly through contaminated animal- and plant-derived products, including poultry, eggs, pork, vegetables and fruits, across the farm-to-fork continuum (Teklemariam et al., 2023). *Salmonella enterica* subsp. enterica serovar Enteritidis (*S. enteritidis*) and *S.* Typhimurium are important epidemic serotypes that have been increasingly associated with antimicrobial resistance (Mulchandani et al., 2024). In recent decades, the overuse of antibiotic has contributed to the emergence and spread of multidrug-resistant (MDR) bacteria. The emergence of MDR *Salmonella* not only causes economic losses but also exacerbates foodborne infections, potentially increasing hospitalization rates and mortality (Hendriksen et al., 2019). Given the limitations of conventional antibiotics in controlling MDR pathogens, it is imperative to develop novel antimicrobial approaches or bacteriolytic agents to prevent MDR *Salmonella* contamination during food production, processing, and storage within the “One Health” principle.

Bacteriophages, the natural bacterial killers, have gained significant attention as promising alternatives to antibiotics due to their specificity, abundance, and minimal side effects. They possess potential application value in the fields of food safety, agriculture, aquaculture, and clinical setting (Ding et al., 2025; Duan et al., 2025; Liu et al., 2025; Zhang et al., 2024). However, the practical implementation of phage therapy remains limited by two major challenges. Firstly, the narrow host range of most phages restricts their effectiveness against diverse bacterial populations, especially in complex contamination scenarios. Secondly, the rapid emergence of phage-resistant bacteria undermines the long-term efficacy of phage-based interventions.

To overcome these limitations, considerable efforts have been devoted to the identification of broad-spectrum phages and the development of phage cocktails (Zhang et al., 2023; Pan et al., 2023). However, the host range of a given phage is fundamentally determined by its receptor recognition specificity. Characterizing the receptor usage of broad-spectrum phages, is essential for understanding the molecular basis of their extended host ranges and provides foundational data for subsequent optimization of phage-based biocontrol strategies.

The therapeutic application of phages is further complicated by the rapid emergence of phage-resistant bacterial strains. Under the long-term phage selection pressure, bacteria have evolved diverse defense mechanisms to evade infection, including receptor modification (Kintz et al., 2015), CRISPR-Cas systems (Al-Shayeb et al., 2020), and abortive infection systems (Sekulovic et al., 2015). Notably, as phage adsorption constitutes the critical initial step of infection, alterations in cell surface receptors represent a primary resistance strategy. Bacterial cell surface components, including lipopolysaccharides (LPS), peptidoglycans, flagella, pili, and some outer membrane proteins, serve as specific adsorption receptors for bacteriophages (Hoyland-Kroghsbo et al., 2017). Bacteria frequently evade phage adsorption and infection through masking, disruption of synthesis, or modification of these receptors via serotype conversion (Hoque et al., 2016). Identification of phage adsorption sites is instrumental not only for understanding host range determinants but also for providing preliminary insights into potential resistance mechanisms, thereby informing strategies to mitigate the emergence of phage resistance.

Bacteriophage JN06 was isolated from the wastewater samples using the MDR *S. enteritidis* strain SMT as the host bacterium. In this study, we systematically characterized its biological properties, genomic features, and environmental stability. The antibacterial efficacy of JN06 was further evaluated both *in vitro* and in food matrices, demonstrating its potential as a biocontrol agent against *Salmonella* contamination. During the characterization process, JN06 was found to lyse multiple *Salmonella* serotypes, indicating a relatively broad host range. Based on this observation, we further conducted a preliminary investigation into its adsorption mechanism using gene knockout and molecular docking approaches. These findings not only provide a comprehensive understanding of the biological characteristics of JN06 but also offer initial insights into the receptor-related basis of its broad host range, thereby supporting its potential application in the control of MDR *Salmonella*.

## 2 Materials and methods

### 2.1 Bacterial strains and phage isolation

*Salmonella enteritidis* strain SMT was isolated from the intestine of a chicken that died of diarrhea and exhibited resistance to ampicillin, amoxicillin, gentamicin, neomycin, and nalidixic acid. All bacterial strains used in this study were listed in Table 1. The strains were cultured in the Tryptic Soy Broth (TSB; Becton, Dickinson and Company, Sparks, MD, USA) at 37 °C with shaking at 180 rpm.

**Table 1:** Host range of phage JN06.

Bacterial genus	Strain name	Origins	Spectrum of drug resistance	Lytic activity
*Salmonella Enteritidis*	SMT	Chicken manure	AMP/AMC/GEN/NEO/NA	+++
	ATCC BAA-708	ATCC	TCY/NA/SXT	+++
	1060b	Chicken breast	AMP/AMC/CTX/TCY/GEN/NEO/FFC/NA/SXT	+++
*Salmonella* Typhimurium	ATCC14028	ATCC	AMP	+++
	ATCC7045	ATCC	AMP/NEO	+++
	CMCC150071	CMCC	AMP/AMC/TCY/CIP	++
	1094b	Chicken breast	AMP/TCY/NA	+++
	2,112	Swine	AMP/ANC/GEN/NA	+++
	1079b	Chicken wing	/	+++
	2,100	Pork	AMP/AMC/TCY/NEO/FFC/CIP/SXT	+++
	1073a	Chicken breast	TCY	+++
	1054b	Chicken breast	TCY/CIP/NA/SXT	++
*Salmonella* Agona	1070b	Chicken breast	AMP/CTX/TCY	+++
	1087a	Chicken breast	AMP/CTX/TCY/FFC/SXT	+
	1087b	Chicken wing	AMP/CTX/TCY/FFC/SXT	+
*Salmonella* Derby	2042a	Pork	AMP/AMC/TCY/GEN/FFC/CIP/NA/SXT	++
	2082b	Pork	AMP/CTX/TCY/CIP/SXT	++
	2054a	Pork	AMP/TCY/CIP	++
*Salmonella* Anatum	1100	Chicken breast	AMP/TCY/SXT	++
	1084a	Chicken breast	CTX/FFC	+
	2089b	Duck	AMP/AMC/TCY/FFC/CIP/NA/SXT	+
*Salmonella* Schwarzengrund	2018	Pork	TCY	+++
*Salmonella* Heidelberg	2081b	Pork	AMP/AMC/TCY	++
	1,086	Chicken wing	AMP/CTX/TCY/FFC	+
*Salmonella* Senftenberg	2071b	pork	AMP/TCY	++
*Salmonella* Kentucky	1080a	chicken breast	AMP/ GEN/ SXT	−
*Salmonella* Rissen	2043a	Pork	TCY	−
*Salmonella* Hadar	2042b	Pork	AMP/AMC/TCY/GEN/FFC/CIP/NA/SXT	−
*Salmonella* Infantis	1068b	Chicken wing	TCY	−
*Escherichia coli*	6–11-8	Chicken wing	AMP/CTX/TCY/GEN/NEO/CIP/NA	−
*Pseudomonas aeruginosa*	9,027	ATCC	AMC/TCY/FFC/SXT	−

ATCC, American Type Culture Collection; CMCC, National Center for Medical Culture Collection.

AMP, ampicillin; AMC, amoxicillin; CTX, cefotaxime; TCY, tetracycline; GEN, gentamicin; NEO, neomycin; FFC, florfenicol; CIP, ciprofloxacin; NA, nalidixic acid; SXT, sulfamethoxazole.

“+++” indicates that the plaque is clear and translucent, “++” indicates that the plaque is slightly turbid, “+” indicates plaque turbidity, and “-” indicates no plaque, respectively.

*Salmonella* phage JN06 was isolated from sewage collected at a broiler breeding site at Jiangxi Agricultural University using an enrichment-based method as previously described (Li et al., 2021), with minor modifications. Briefly, CaCl_2_ (1 mmol/L) was added to the sewage samples, which were then incubated at 37 °C for 2 h. After centrifugation (3,000 × g, 10 min, 4 °C), the supernatant was filtered through the 0.22 μm sterile filter. This was mixed with *S.* Enteritidis SMT (10^8^ CFU/mL) in TSB medium (1:1 ratio), followed by 12 h incubation at 37 °C with shaking at 220 rpm. Following enrichment, the culture was centrifuged and filtered (0.22 μm) to obtain the phage-containing supernatant. The presence of phages was confirmed by the spot tests. Phage suspensions were serially diluted in SM buffer (150 mM NaCl, 50 mM Tris-base, 10 mM MgCl_2_^.^6H_2_O and 2 mM CaCl_2_). And individual plaques were isolated using the double-agar method (Ongenae et al., 2022). Single plaques were picked and purified through at least three successive rounds of plating to ensure homogeneity. High-titer phage stocks were obtained by propagation with the host strain and stored at 4 °C for subsequent experiments.

### 2.2 Transmission electron microscopy (TEM)

The prepared phage (10^10^ PFU/mL) were transferred to a carbon-coated copper grid (Beijing Daji Technology Co., Ltd.) and allowed to float for 3–5 min before being retrieved. Excess liquid was absorbed from the edge of the copper grid using a filter paper strip. After slightly drying, it was placed in the negative staining solution of phosphotungstic acid for 2 min. Excess staining solution was absorbed, and it was placed under a drying lamp for drying. The morphology of the phages was observed under a Hitachi HT7800 transmission electron microscope (Tokyo, Japan) at an accelerating voltage of 60 kV.

### 2.3 Determination of the host range

The host range of phage JN06 was assessed by spotting tests. Briefly, the test strains of *Salmonella*, *Escherichia coli* and *Pseudomonas aeruginosa* (Table 1) were cultivated in TSB medium to the logarithmic phase, and 100 μL of the each was added to 7 mL of TSB medium containing 0.75% agar. After thorough mixing, it was promptly poured onto TSB agar plates. 10 μL phage suspension (10^8^ PFU/mL) was dropped onto the agar plates, and an equal volume of SM buffer was dropped in another area as a negative control. The plates were left to stand for 2 h in a 37 °C constant temperature incubator and then inverted for 12–16 h of incubation. The lysis performance of phage JN06 was evaluated by the morphology of the plaques formed on the lawns.

### 2.4 Biological characteristics of JN06

#### 2.4.1 The optimal multiplicity of infection

The optimal multiplicity of infection (MOI) was determined as previously described (Zhang et al., 2023). 100 μL (with MOIs of 100, 10, 1, 0.1, 0.01, 0.001, and 0.0001, respectively) and 100 μL *S. enteritidis* SMT (10^8^ CFU/mL) were added to 10 mL of TSB medium. After incubation at 37 °C for 15 min, the culture was transferred to a constant temperature shaker incubation for 5 h at 37 °C and 220 rpm. The culture was centrifuged at 4 °C and 12,000 × g for 10 min, and the supernatant was filtered and sterilized using a 0.22 μm sterile filter. The phage titers obtained with different MOIs were determined using the double-agar overlay TSB agar plates. The MOI that resulted in the highest phage titer was defined as the optimal multiplicity of infection (MOI). The experiment was conducted in duplicate with three replicates each.

#### 2.4.2 One-step growth curve

The experimental method for the one-step growth curve referred to the method of Wang et al. and was slightly modified (Wang et al., 2010). *S.* enteritidis SMT (10^8^ CFU/mL) and the phage suspension with the optimal MOI were thoroughly mixed in 10 mL of TSB medium and incubated at 37 °C for 15 min for infection. Subsequently, the mixture was centrifuged at 4 °C and 12,000 × g for 2 min to eliminate the unabsorbed free phages. After washing with TSB, the resulting pellet was resuspended in TSB and added to 500 mL of TSB culture medium. The culture medium was incubated at 37 °C and 220 rpm. 500 μL samples were collected at 0, 2, 4, 6, 8, 10, 15, 20, 25, 30, 35, 40, 50, 60, 80 min post-infection, respectively. These samples were centrifuged at 4 °C and 12,000 × g for 30 s, the supernatant was aspirated and filtered through 0.22 μm sterile filter, and then the titer of phage was determined using a double-layer plate. The experiment was conducted in duplicate with three replicates each.

#### 2.4.3 Determination of the stability of phage at different pH and temperatures

To assess the temperature stability of phage JN06, 1 mL phage suspension (10^9^ PFU/mL) was taken and added to sterilized 2-mL centrifuge tubes at 4 °C, 25 °C, 37 °C, 50 °C, 60 °C, 70 °C, and 80 °C for 15 min, 30 min, and 60 min, respectively. Similarly, for the assessment of pH stability, 100 μL of phage was added to 900 μL of SM buffer at different pH values, and then incubated at 37 °C for 1 h. The pH of the SM buffer ranging from 2 to 13 was adjusted by 1 M HCl or 1 M NaOH. Subsequently, the phage titer was measured using the double-agar overlay TSB agar plates. The experiment was conducted in duplicate with three replicates each.

### 2.5 Genomic analysis and phage characterization

Phage genomic DNA was extracted following concentration and purification of phage particles. Briefly, polyethylene glycol 8,000 (PEG8000, Solarbio, Beijing, China) was added to the phage suspension at a final concentration of 10%, followed by incubation at 4 °C until fully dissolved. The mixture was then centrifuged at 12,000 × g for 15 min at 4 °C, and the resulting pellet was resuspended in SM buffer to obtain the concentrated phage suspension. To eliminate residual host nucleic acids, the suspension was treated with DNase I and RNase A prior to DNA eatraction. Phage genomic DNA was extracted using the TIANamp Virus DNA/RNA kit (Tiangen, Beijing, China) according to the manufacturer’s instructions. Whole-genome sequencing was performed using the Illumina MiSeq platform (San Diego, California, USA) by Megagene Technology Co. Ltd. (Guangzhou, China). Raw reads were quality-filtered and assembled using SPAdes v3.15.2 (Prjibelski et al., 2020) with default parameters, and assembly quality was evaluated using QUAST v4.4 (Gurevich et al., 2013). Genome annotation was performed using the RAST.^1^ To improve annotation reliability, predicted open reading frames (ORFs) were further manually curated based on sequence homology searches against the NCBI database and conserved domain analysis. Only annotations with consistent functional prediction were retained. Transfer RNA genes were identified using tRNAscan-SE^2^ (Schattner et al., 2005). Genome visualization was performed using the CGView server^3^ (Grant and Stothard, 2008). Potential virulence factors and antibiotic resistance genes were screened using VirulenceFinder^4^ and ResFinder,^5^ respectively, with default thresholds. Lysogeny-related genes were identified based on homology searches against annotated phage genomes and conserved domain analysis.

To determine the taxonomy of the phage, download the sequences of the terminase large subunit belonging to different phages from the NCBI database according to the International Committee on Taxonomy of Viruses (ICTV) classification report. Multiple sequence alignment was performed, and a phylogenetic tree was constructed using the Neighbor-joining method in MEGA v7.0 with 1,000 bootstrap replicates to assess branch sopport (Kumar et al., 2016). The evolutionary distances were computed using the Poisson correction model. Average nucleotide identity (ANI) analysis was conducted between JN06 and 29 reference phage genomes obtained from NCBI using OrthoANI (OAT) with default settings. ANI values were visualized as a heatmap using TBtools-II. An ANI threshold of approximately 95% was used as a reference for species-level relatedness.

Comparative genomic analysis was performed using Easyfig to visualize genome synteny. In addition, multiple sequence alignment of tail fiber and tail spike proteins from JN06 and selected closely related phages (based on ANI results) was conducted using MEGA v7.0.

### 2.6 Assessment of the antibacterial activity of phage JN06 *in vitro*

#### 2.6.1 The antibacterial performance of JN06 in *vitro*

The ability of phage JN06 to inhibit the growth of *S. enteritidis* SMT was evaluated using optical densitometry. Briefly, 1 mL of phage JN06 (with MOI values of 100, 10, 1, 0.1, 0.01, 0.001, and 0.0001) was mixed with 1 mL of *S. enteritidis* SMT (10^8^ CFU/mL), and then added to 100 mL of TSB liquid culture medium. The mixture was incubated at 37 °C in a shaking incubator for 24 h. Samples were collected every 4 h, and the 600 nm reading was measured to monitor the growth of *S. enteritidis* SMT. In the positive control group, the phage was replaced by an equal volume of SM buffer. The experiment was repeated three times with three biological replicates each time.

#### 2.6.2 Evaluation of the inhibitory and clearance effects of JN06 on biofilms

The antibiofilm effect of JN06 was evaluated using 12-well flat-bottomed polystyrene. For the biofilm inhibition assay, overnight cultures of *S. enteritidis* SMT were diluted at 1:100 (v/v) with LB medium to approximately 10^7^ CFU/mL. Then, 300 μL of the diluted bacterial suspension was added to a 12-well flat-bottomed polystyrene plates. Phage lysate was immediately added at a final titer of 10^8^ or 10^9^ PFU/mL to assess its effect on biofilm formation. The group without phage addition served as the positive control. The 12-well flat-bottomed polystyrene were incubated statically at 37 °C for 48 h. After incubation, planktonic cells were carefully removed, and the wells were washed twice with sterile PBS to eliminate non-adherent bacteria. The number of viable attached cells was determined by resuspending the biofilm, followed by 10-fold serial dilution and plating on LB agar. Biofilm biomass was quantified by crystal violet staining. Briefly, wells were stained with 1% crystal violet for 10 min, washed with distilled water to remove excess dye, and air-dried. The bound dye was solubilized using 33% acetic acid, and the absorbance was measured at 595 nm using the ELISA microplate reader (Biotek, VT, USA) (Li et al., 2024). This experiment was conducted in triplicate.

For the biofilm eradication assay, biofilms were first established by incubating bacterial suspensions under the same conditions for 48 h. After removal of planktonic cells and washing with PBS, pre-formed biofilms were treated with 3 mL of LB medium containing phage JN06 at final titers of 10^8^ or 10^9^ PFU/mL, LB medium alone as a control, and incubated at 37 °C for an additional 24 h. Thereafter, residual biofilm biomass and viable attached cells were quantified as described above. Residual biofilm was quantified by absorbance measurements as described previously.

The lytic effect of phage on the prevention of biofilm formation and its eradication was evaluated by Confocal Laser Scanning Microscopy (CLSM). Bacteria viability and biofilm distribution after phage coincubation/treatment was determined by CLSM after staining cells with Syto 9 (488 nm/500–540 nm) and propidium iodide (PI) (561 nm/600–650 nm) following manufacturer protocols (Live/dead BacLight Bacterial Viability Assay Kit, Molecular Probes, Life Technologies). Samples were analyzed by the microscope Olympus FV3000 (Olympus, Tokyo, Japan) using a 20 × objective.

### 2.7 Application of phage JN06 as effective food biocontrol on lettuce

The bactericidal activity of JN06 on artificially contaminated lettuce was evaluated as previously described (Zhu et al., 2026). Fresh lettuce was purchased from a supermarket in Nanchang, China. Lettuce leaves were cut into square pieces measuring 2 × 2 cm, washed with sterile water, and surface-sterilized by spraying with 75% ethanol, and exposed to ultraviolet light for 30 min to minimize background microbial interference. The concentrations employed for simulating *Salmonella* contamination and phage treatment were referenced from the prior publications of our research group. Lettuce sections were inoculated with *S. enteritidis* SMT at approximately 10^4^ CFU/cm^2^ (100 μL per sample) and allowed to air-dry at room temperature for 30 min to facilitate bacterial attachment. Subsequently, 100 μL of purified phage suspension (10^8^ PFU/cm^2^) was applied to each sample. The relatively high phage concentration was selected to ensure effective phage–bacteria contact under surface-associated conditions (Pan et al., 2023; Zhang et al., 2023). Control samples received sterile saline instead of phage. Treated samples were incubated at either 4 °C or 25 °C for up to 3 days, and samples were collected at 24 h intervals. At each sampling point, bacteria were recovered by adding 4 mL of elution buffer (0.025% SDS and 0.85% saline) followed by vortexing for 2 min. The resulting suspension was serially diluted (10-fold), and viable bacterial counts were determined by plating on XLD agar. This experiment was conducted in triplicate.

### 2.8 Screening and identification of phage-resistant strains

Phage-resistant mutants were isolated to investigate the potential mechanisms of resistance and to provide a basis for subsequent receptor identification. The double-layer plate method was used to screen for resistant strains. Briefly, *S.* Enteritidis SMT was inoculated into TSB medium (1%, v/v) and cultured at 37 °C with shaking (220 rpm) until OD_600_ reached approximately 1.2. Then, 100 μL of bacterial culture was mixed with 100 μL of phage suspension (MOI = 100) and combined with 7 mL of molten TSB soft agar (0.75%). The mixture was overlaid onto LB agar plates and incubated at 37 °C overnight. Colonies appearing on the plates were considered putative phage-resistant mutants. 20 single colonies from each plate were randomly selected and subjected to five successive rounds of streak purification on LB agar plates. Phage resistance was confirmed by spot assay. To evaluate phenotypic changes associated with resistance, purified mutants were cultured on Columbia blood agar plates (Hopebio, Qingdao, China) and colony morphology was recorded after incubation at 37 °C for 12–16 h.

To further assess potential alterations in surface antigens, an agglutination assay was performed using an O_9_ mAB (a monoclonal antibody that specifically recognizes O_9_ antigen). Briefly, bacterial colonies were suspended in either O_9_ mAB (200 μL, 50 μg/mL; BioChek) or physiological saline (200 μL, 9 mg/mL) and gently mixed. If no agglutination was observed within 1 min, it indicated that the O-antigen of the colony had undergone alteration.

### 2.9 Identification of the host receptor for phage JN06

#### 2.9.1 Identification of the phage receptor type

To determine whether the phage receptor is associated with surface polysaccharides or proteins, bacterial cells were treated with periodate (IO_4_^−^) or proteinase K, respectively, as previously described (Kiljunen et al., 2011). Briefly, IO_4_^−^ (100 mM) prepared in sodium acetate buffer (50 mM, pH 5.2) was added to *S*. Enteritidis SMT suspension (3 × 10^9^ CFU/mL) and incubated at 25 °C in the dark for 2 h. For proteinase K treatment, bacterial suspensions were incubated with proteinase K (0.2 mg/mL) at 37 °C for 3 h. Following treatment, cells were washed with PBS, and phage adsorption assays were immediately performed. Phage JN06 (10 μL, 1 × 10^6^ PFU/mL) was mixed with treated bacterial suspensions and incubated at 37 °C with shaking (180 rpm) for 15 min. The mixtures were centrifuged (12,000 × g, 5 min, 4 °C), and the supernatant was filtered (0.22 μm). The residual phage titer in the supernatant was determined using the double-layer agar method. Phage adsorption was calculated as [(initial titer − residual titer in the supernatant) / initial titer] × 100% (Le et al., 2013).

#### 2.9.2 Effect of LPS on phage adsorption

To further evaluate the role of LPS in phage adsorption, LPS was extracted from *S. enteritidis* SMT using an LPS extraction kit (Bestbio Biotechnology), extensively dialyzed against Milli-Q water (4 changes for 2 days) and lyophilized. Phage JN06 (500 μL of 10^6^ PFU/mL) were mixed with LPS solution (500 μL of 50 mg/mL) and incubated at 37 °C for 1 h. Control samples were prepared using PBS instead of LPS. The remaining phage titer was determined using the double-layer agar method to assess whether LPS interfered with phage adsorption.

### 2.10 Role of the O-antigen in phage adsorption

#### 2.10.1 Construction of O-antigen deficiency strain

The *rfbP* is a key gene for synthesizing O antigen. To validate the role of O-antigen in phage adsorption, a *rfbP* deletion mutant was constructed using the *λ* Red recombination system as previously described (Wang et al., 2017). Refer to the *rfbP* gene information for *S. enterica* in the public database NCBI, primers rfbP-HF and rfbP-HR containing homologous arms were synthesized. Using pKD3 as a template, PCR amplification was carried out to construct the *rfbP* targeting fragment. The targeting fragment was then transformed into *S. enteritidis* SMT harboring pKD46. Following verification by PCR analysis and gene sequencing, the *rfbP* gene deletion strain SMT-Δ*rfbP* was successfully obtained. For the complementation strain, we constructed pBAD33: C*rfbP* followed a previously described method (Li et al., 2023). Subsequently, the recombinant plasmid was transformed into the mutant strain SMT-Δ*rfbP*. Monoclonal colonies were selected and confirmed by PCR. Positive colonies were selected for gene sequencing verification. The complement mutant was named SMT-C-Δ*rfbP*. The strains, plasmids and PCR primers employed in this study are presented in Supplementary Tables S1, S2.

#### 2.10.2 LPS preparation and SDS-PAGE analysis

LPS from *S. enteritidis* strains was extracted using an LPS extraction kit (Bestbio Biotechnology). Purified LPS was assessed following separation on a 12% Sodium dodecyl sulfate-polyacrylamide gel and staining using a PAGE Gel Silver Staining Kit (Solarbio).

#### 2.10.3 Phage infectivity and adsorption assays

Phage infectivity was assessed using spot assays with serially diluted phage suspensions. For adsorption assays, phage (10^6^ PFU/mL) was mixed with bacterial cultures at MOI 0.001 and incubated at 37 °C. After 10 min, the supernatant was collected, and the phage titer was determined. Adsorption rate was calculated as follows: adsorption rate (%) = (phage titer in SM buffer - phage titer in supernatant) / (phage titer in SM buffer) × 100%.

### 2.11 Molecular docking analysis

To explore the molecular basis of phage-host interaction, molecular docking analysis was performed between predicted O-antigen structures and phage tail proteins. The structure of Gal-initiated O-antigen has not yet been reported. To obtain predicted O-antigen model, we used the *S.* Typhimurium O-antigen (PubChem CID: 46878556) and *Escherichia coli* serotype O86 O-antigen (PubChem CID: 91862843) as a reference and performed structural simulation using GLYCAM-Web.^6^ The homology model of tail fiber protein and tail spike protein was obtained from Swiss-Model.^7^ Docking simulations were performed using AutoDock Vina (Version 1.1.2; Scripps Research, CA, USA). Binding poses were ranked by binding free energy (ΔG, kcal/mol). Protein-ligand interactions were analyzed using PyMOL software (Version 2.5.4; Schrӧdinger, LLC, NY, USA) and Discovery Studio (DS4.5, Accelrys Inc).

### 2.12 Statistical analysis

Statistical analyses were conducted using SPSS Statistics v27.0.1.0. Each experiment was replicated three times, and the results are expressed as means ± standard deviation (SD). Mean comparisons were performed by one-way analysis of variance (ANOVA), followed by Dunnett’s *t*-test or Duncan’s test. The significance level was set to 0.05. Graphical data processing was carried out using GraphPad Prism 10.0 and Origin 2021.

## 3 Results

### 3.1 Isolation and morphology analysis of phage JN06

**Figure 1:**
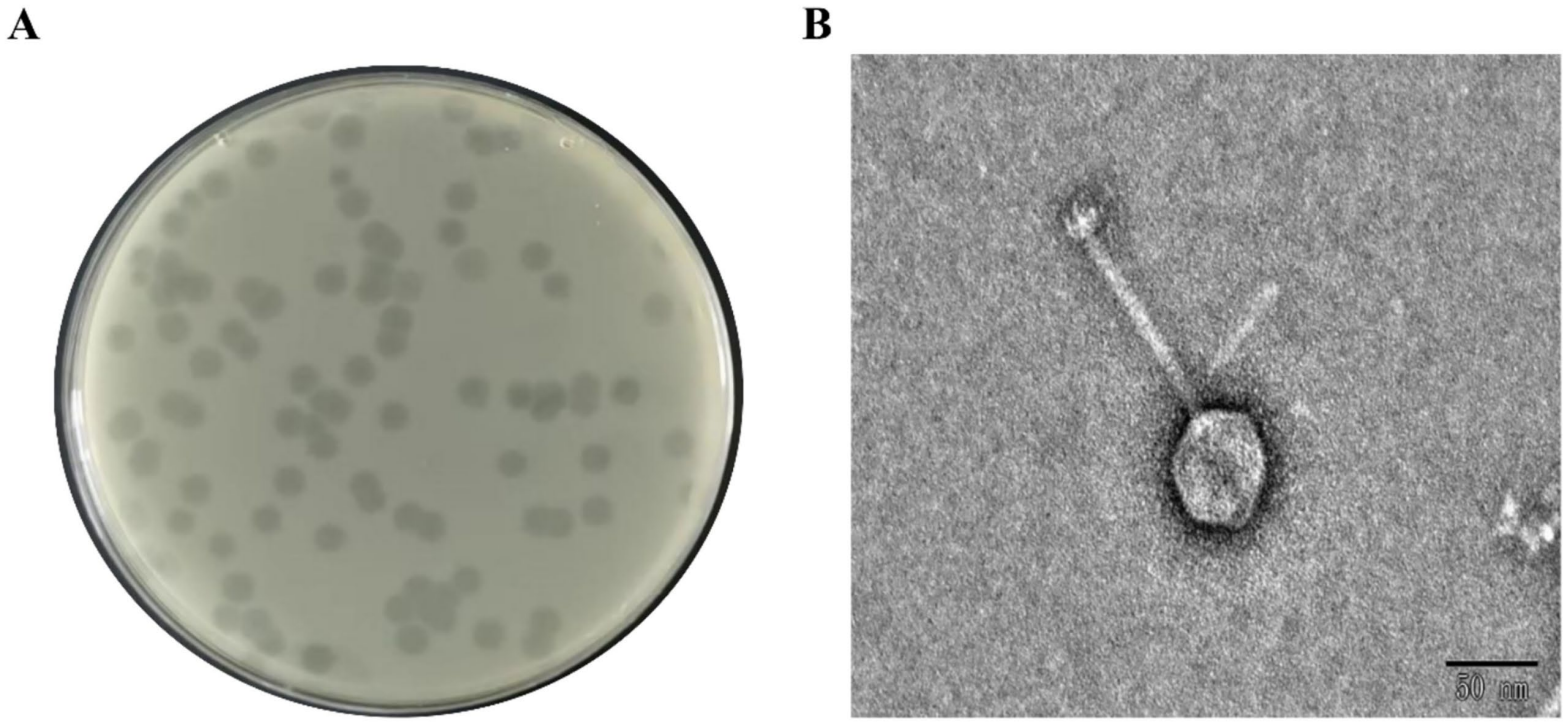
Morphology characterization of phage JN06. **(A)** Plaque morphology of phage JN06 on the double-layer agar plate using *S. enteritidis* SMT. **(B)** Transmission electron micrographs of JN06. Scale bar: 50 nm.

The bacteriophage JN06 was isolated from the wastewater samples using *S. enteritidis* SMT (isolated from chicken manure) as the host strain. It formed clear and transparent circular spots with a diameter of approximately 2 mm on the bacterial lawn of *S. enteritidis* SMT (Figure 1A).

Transmission electron microscopy revealed that phage JN06 possessed a polyhedral head and a long and non-contractile tail, consistent with morphological characteristics of the class *Caudoviricetes*. The head diameter of JN06 was approximately 50 ± 0.2 nm, and the tail length was about 110 ± 0.2 nm (Figure 1B).

### 3.2 Host range of phage JN06

The host range of phage JN06 was determined by using *Salmonella*, *Escherichia coli* and *Pseudomonas aeruginosa*. Phage JN06 could infect 23 of the 27 tested *Salmonella* strains, the infection rate reached as high as 85.18% (Table 1). As shown, JN06-dependent killing was observed in previously archived and isolated foodborne *Salmonella* (mainly from livestock or poultry-derived products). Collectively, JN06 was capable of lysing the *S. enteritidis*, *S.* Typhimurium, *S.* Agona, *S.* Derby, *S.* Anatum, *S.* Schwarzengrund, *S.* Heidelberg and *S.* Senftenberg, showed lytic activity against multiple serotypes.

### 3.3 Biological characteristics analysis

**Figure 2:**
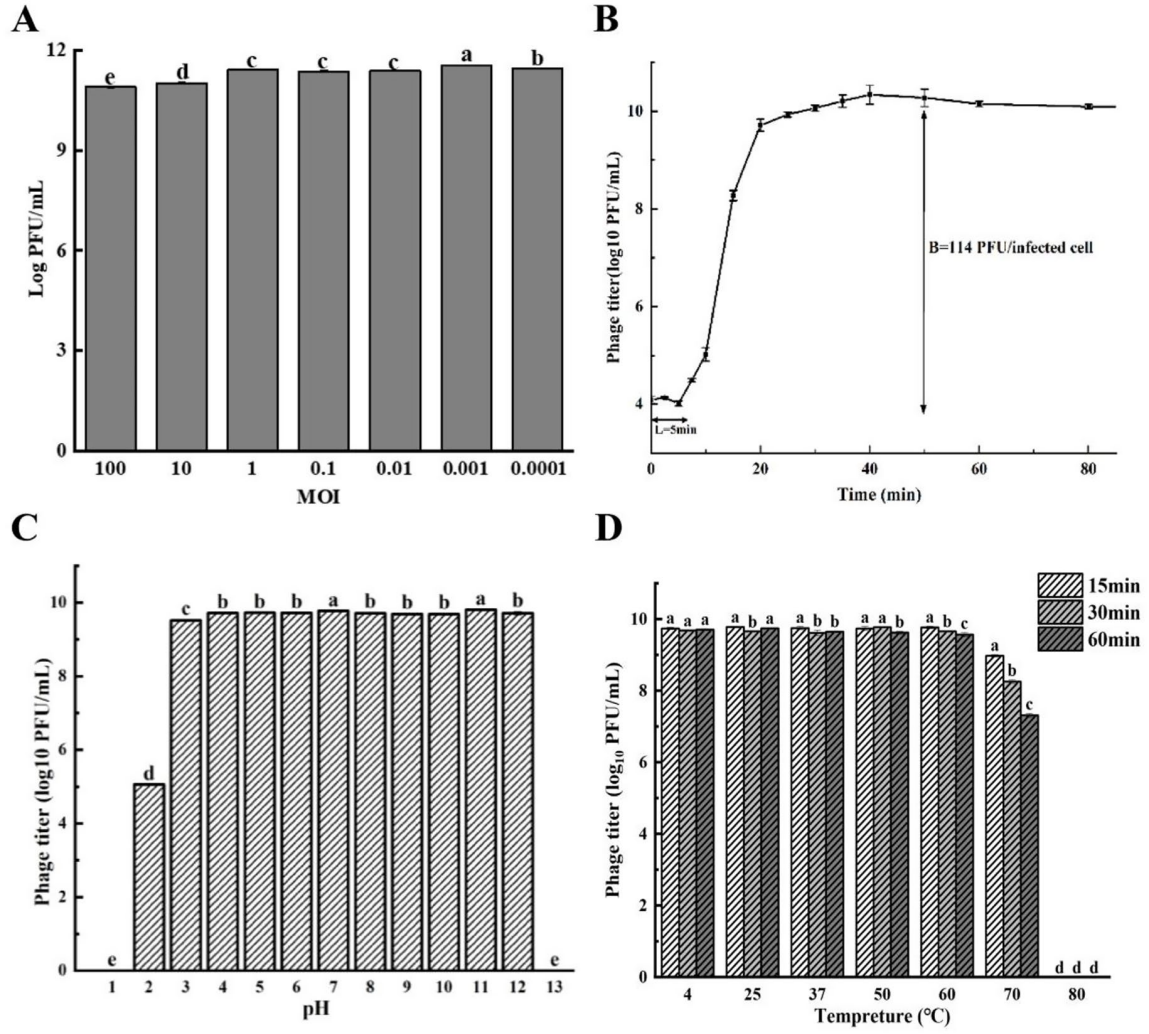
Biological characteristics of phage JN06. **(A)** Multiplicity of infection (MOI) of phage JN06. **(B)** One step growth curve. Phage JN06 had a latent period of approximately 6 min, an exponential growth period of 20 min, and a final plateau phase from 25 min onward. The estimated burst size of JN06 was 131 PFU/infected cell. The experiment was repeated three times, and results are expressed as mean ± SD. **(C)** Stability of JN06 under different pH conditions (pH 1–13). **(D)** Thermal stability of JN06 at different temperatures (4–80 °C). Results were presented as the mean ± SD, *n* = 3/group; mean comparisons were performed using one-way ANOVA, followed by Duncan’s test; a–e: different letters represent significant differences (*p* < 0.05) between groups.

As demonstrated in Figure 2A, the highest phage titer was obtained at an MOI of 0.001 under the tested conditions. Remarkably, the experimental results of the one-step growth curve demonstrated that the incubation period of JN06 was exceptionally short, and a phage propagation was determined with a lag period of only 6 min alongside a burst size of 131 PFU/infected cell (Figure 2B).

The pH and temperature stability of phage JN06 was evaluated. For pH stability, JN06 remained highly stable over a wide pH range (pH 3–12), with no significant reduction in titer. Even under highly acidic conditions (pH 2), approximately 51.58% of phage activity was retained (Figure 2C). For temperature stability, JN06 maintained stable titers at 4 °C, 25 °C, and 37 °C across all tested time points (15, 30, and 60 min). In contrast, phage activity decreased at elevated temperatures. At 70 °C, a progressive decline in phage titer was observed with increasing incubation time, with a reduction of approximately 2.43 log₁₀ PFU/mL after 60 min (Figure 2D).

### 3.4 Genomic analysis of phage JN06

**Figure 3:**
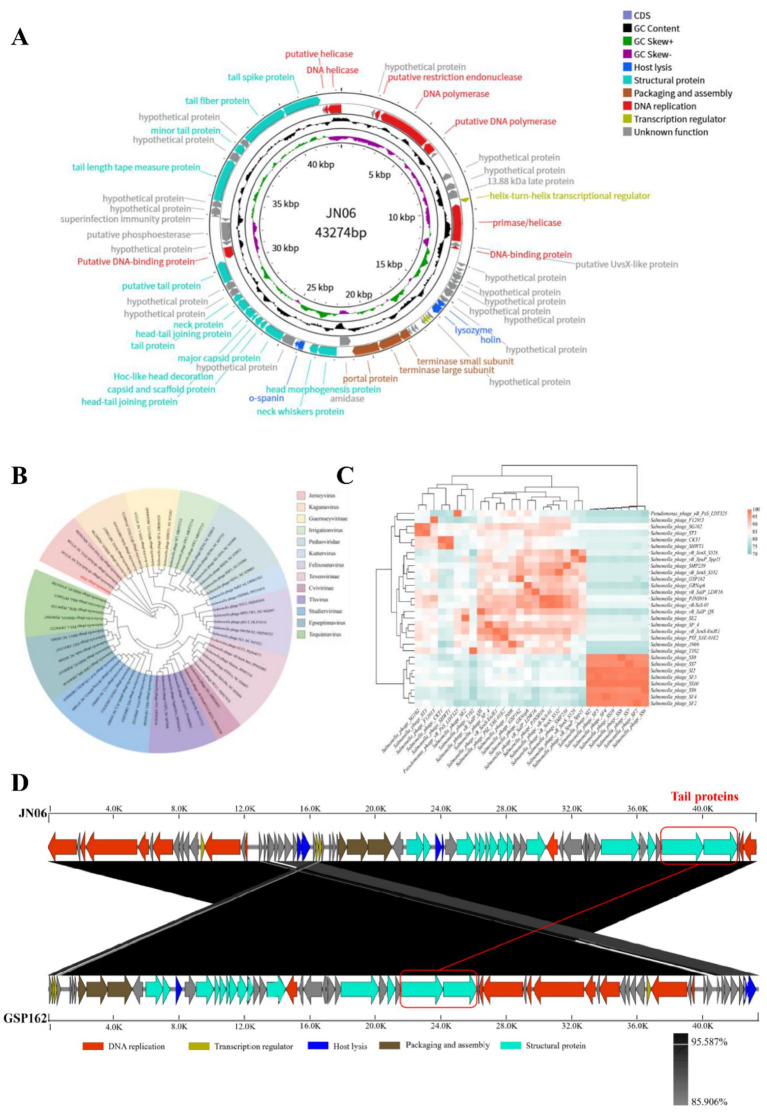
Genomic characterization of JN06. **(A)** Circular genome map of JN06 generated using CGView Server (from the inside to outer circle): (1) GC skew plot. (2) G + C % content. (3) ORFs transcribed in the clockwise or the counterclockwise direction. **(B)** Phylogenetic analysis based on the terminase large subunit, constructed using Neighbor-joining method in MEGA v7.0, showing the taxonomic position of JN06 within in class *Caudoviricetes*. **(C)** Heatmap of average nucleotide identity (ANI) values among JN06 and 29 reference phage genomes, calculated using OrthoANI and visualized with TBtools-II. **(D)** Genome collinearity comparison between JN06 and *Salmonella* phage GSP162 generated using Easyfig.

The phage JN06 was identified as a double-stranded DNA phage, with a genome length of 43,274 bp and with GC content of 50.27% (Figure 3A), the genomic information of this phage has been uploaded to NCBI database and obtained an accession number (PQ411278). A total of 66 ORFs were predicted, of which 37 were assigned putative functions, while the remaining ORFs encoded hypothetical proteins. Functional annotation revealed genes associated with host lysis, structural proteins, packaging and assembly, DNA replication, and transcriptional regulation. No genes related to lysogeny, virulence, or antibiotic resistance were identified.

### 3.5 Phylogenetic analysis and average nucleotide identity (ANI) of phage JN06

To understand the phylogenetic relationship of phage JN06, phylogenetic analysis was performed using the highly conserved terminase large subunit gene within phage JN06. 58 terminase large subunit genes of phages were retrieved from the NCBI database for the construction of a phylogenetic tree. According to the classification criteria of the ICTV, phage JN06 belonged to the genus *Jerseyvirus* of class *Caudoviricetes* (Figure 3B). To further understand the homology of phage JN06 with other phages of the genus *Jerseyvirus*, 29 phages belonged to the genus *Jerseyvirus* were selected in this study for whole genome average nucleotide identity analysis. As shown in Figure 3C, phage JN06 exhibited the highest homology (93.28%) with *Salmonella* phage GSP162 and the lowest homology (78.04%) with *Salmonella* phage SF2. Notably, the ANI values between JN06 and all reference phages were below the commonly accepted species demarcation threshold (~95%), indicating that JN06 is genetically distinct from previously reported *Jerseyvirus* phages.

The comparison of linear genome maps between JN06 and GSP162 showed some differences in their lytic enzymes, but the tail proteins were highly similar (Figure 3D). Subsequently, we performed a multiple-sequence alignment analysis on the tail fiber protein and tail spike protein of JN06 and those of seven other bacteriophages. These results indicated that the tail fiber proteins of JN06 and GSP162 exhibited a high degree of similarity. Specifically, the similarity between the tail fiber proteins of them reached as high as 99% (Supplementary Figure S1), with only two amino acid discrepancies.

### 3.6 Antibacterial activity of phage JN06

To investigate the antibacterial capacity of phage JN06, this study examined the lysis efficacy of phage on host bacteria at different MOIs (0.0001 ~ 100). Phage treatment delayed bacterial growth (Supplementary Figure S2). Compared with the control group, the growth of bacteria in the phage treatment groups were significantly inhibited at the early stage (4–12 h). At 8 h, the OD₆₀₀ value of the control group reached approximately 1.3, while that of the phage treatment group remained close to the baseline level, indicating a growth delay of about 4 to 8 h. At 12 h, the OD₆₀₀ value of the treatment group was still about 0.3 to 0.5 lower than that of the control group, especially in the treatment groups with MOI > 0.1.

### 3.7 Anti-biofilm activity of phage JN06

**Figure 4:**
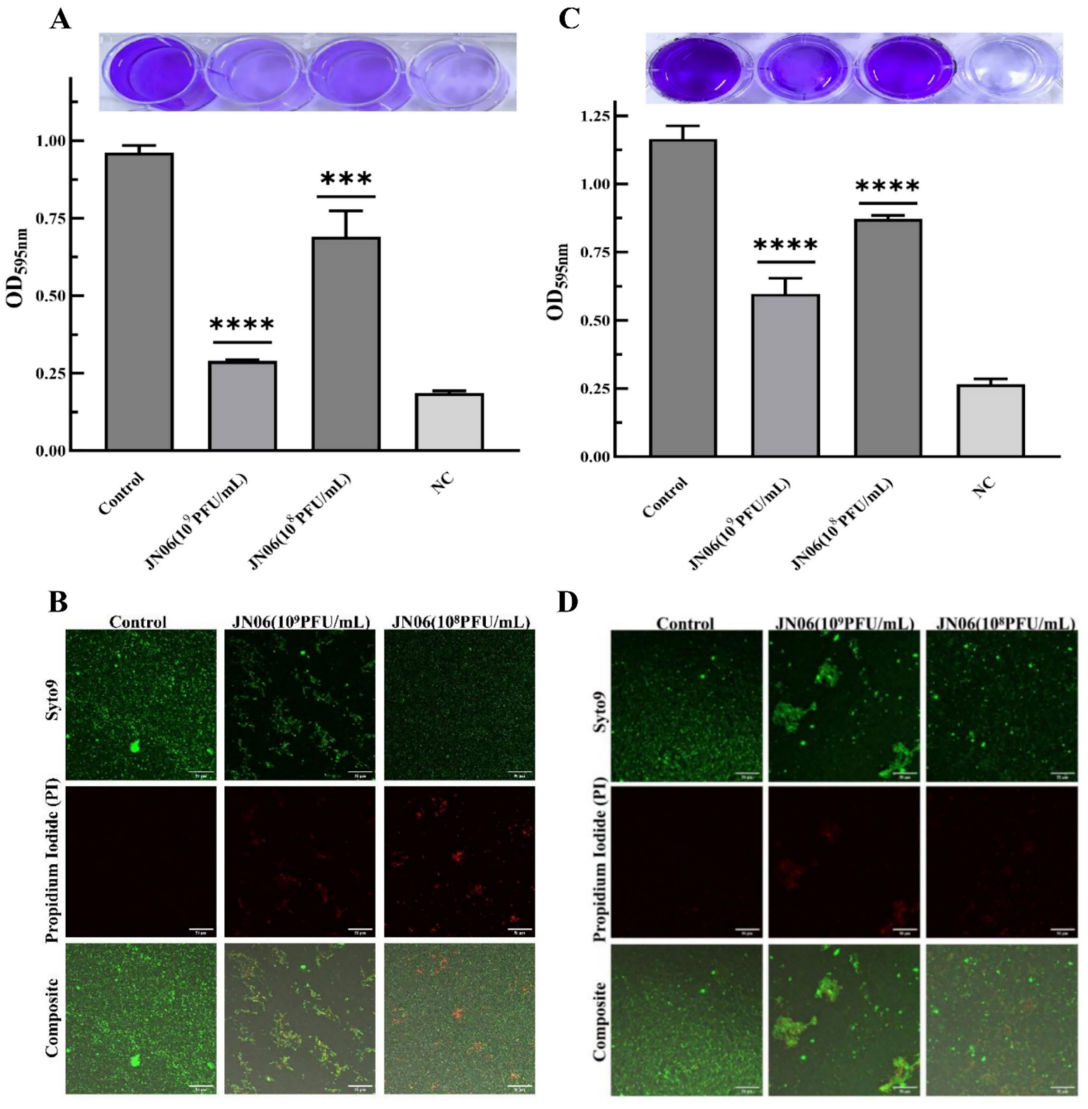
Antibiofilm activity of phage JN06 against *S. enteritidis* SMT. **(A,B)** Inhibition of biofilm formation by JN06. **(C,D)** Disruption of preformed biofilms by JN06. Biofilm biomass was quantified by crystal violet staining **(A,C)**, and biofilm structure and cell viability were visualized by crystal violet staining and CLSM **(B,D)**. Live cells were stained green (Syto 9), and dead cells were stained red (propidium iodide). NC, Negative control (no bacterial inoculation). Data were presented as mean ± SD; ***p < 0.001, ****p < 0.0001; Scale bar: 50 μm.

The effect of JN06 on the biofilm formation of *S. enteritidis* SMT was evaluated using 12-well plates. For biofilm formation, different concentrations (10^9^ or 10^8^ PFU/mL) of phage were incubated with *S. enteritidis* SMT for 48 h. Compared with the control group, phage treatment significantly reduced biofilm biomass, with inhibition rates of 86.58 and 20.79% at 10^9^ and 10^8^ PFU/mL, respectively (Figure 4A). Viable-cell count results showed that phage treatment reduced the viable bacterial population by 2.23 and 0.99 log₁₀ CFU/mL at 10^9^ and 10^8^ PFU/mL, which corresponded to a significant inhibition of biofilm formation (Supplementary Figure S3). For established biofilms, at 48 h of biofilm formation, mature biofilms were treated with different concentrations (10^8^ or 10^9^ PFU/mL) of JN06 for 24 h. As shown in Figure 4C and Supplementary Figure S3, phage JN06 could significantly reduce established biofilms. CLSM further confirmed these findings, showing reduced biofilm density and increased proportions of non-viable cells in phage-treated groups (Figures 4B,D).

### 3.8 Antibacterial activity of phage JN06 on lettuce

**Figure 5:**
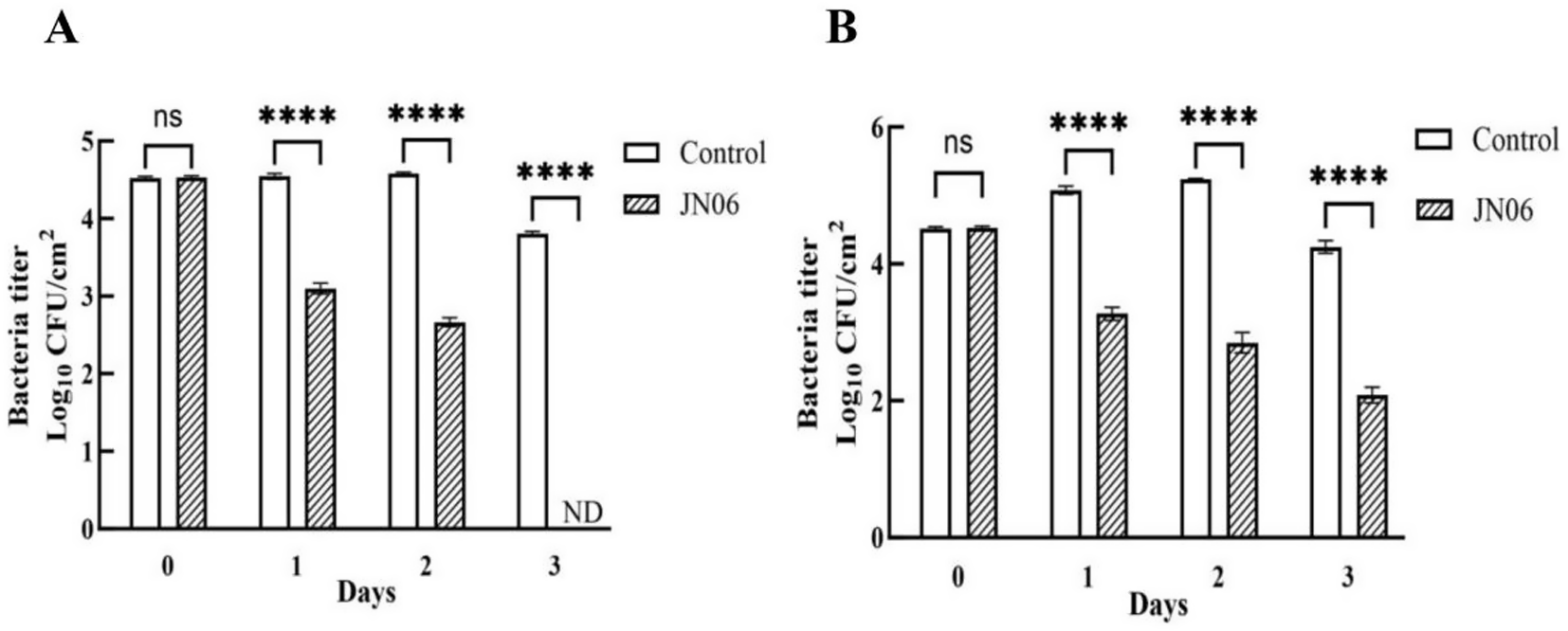
Bacterial inhibition of phage JN06 on lettuce. Bacterial counts on lettuce stored at **(A)** 4 °C and **(B)** 25 °C following phage treatment. Data were presented as mean ± SD. “ND” represented that bacterial counts were below the detection limit (< 10 CFU/cm^2^); ****p < 0.0001.

The ability of phage JN06 to reduce *S.* Enteritidis SMT contamination on lettuce during storage at 4 °C and 25 °C was assessed (Figures 5A,B). At 4 °C, the bacterial population decreased by 1.43, 1.86, and 4.53 log₁₀ CFU/cm^2^ after 1, 2, and 3 days of phage treatment, respectively. At 25 °C, reductions of 1.26, 1.68, and 2.45 log₁₀ CFU/cm^2^ were observed after 1, 2, and 3 days, respectively. Overall, phage JN06 effectively reduced *S. enteritidis* SMT on lettuce under both storage conditions.

### 3.9 Colony morphology and agglutination analysis of phage-resistant strains

Phage-resistant mutants were isolated and characterized to investigate potential resistance-associated phenotypic changes. Compared with the wild-type strain, which exhibited smooth and rounded colonies, all resistant mutants displayed varying degrees of wrinkled colony morphology. Agglutination assays further revealed that the wild-type strain showed clear agglutination with O9 mAB, whereas none of the four resistant mutants exhibited agglutination (Supplementary Figure S4). These results indicated that the O-antigen of the phage-resistant mutants had undergone alterations.

### 3.10 Identification of the adsorption receptor for phage JN06

**Figure 6:**
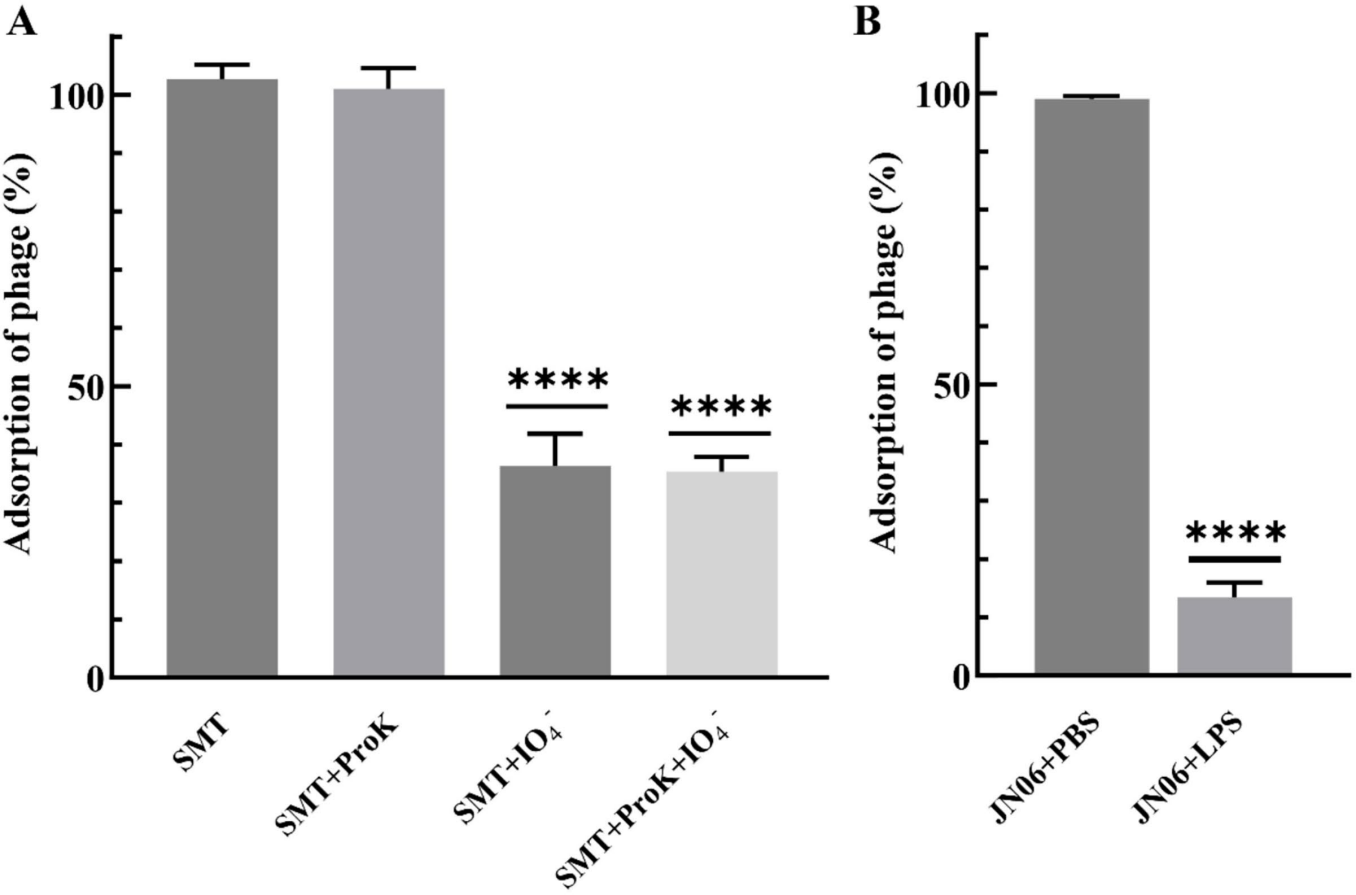
Identification of the adsorption receptor of phage JN06. **(A)** Adsorption efficiency of JN06 to *S. enteritidis* SMT after treatment with periodate (IO_4_^−^) or proteinase (ProK). **(B)** Effect of purified LPS on phage adsorption capacity. Data were presented as mean ± SD; ****p < 0.0001.

In order to identify candidate receptor of phage JN06, *S. enteritidis* SMT was treated with IO_4_^−^ and/or proteinase K prior to adsorption assays. Proteinase K treatment did not significantly affect phage adsorption compared to untreated cells. In contrast, IO_4_^−^ treatment resulted in a marked reduction in adsorption efficiency. Combined treatment with proteinase K and IO_4_^−^ showed no additional effect beyond IO_4_^−^ treatment alone (Figure 6A). Furthermore, incubation of phage JN06 with purified LPS (50 mg/mL) significantly reduced its adsorption capacity to *S. enteritidis* SMT (Figure 6B). These results indicated that LPS serves as the receptor for JN06.

### 3.11 Role of O-antigen in phage adsorption

**Figure 7:**
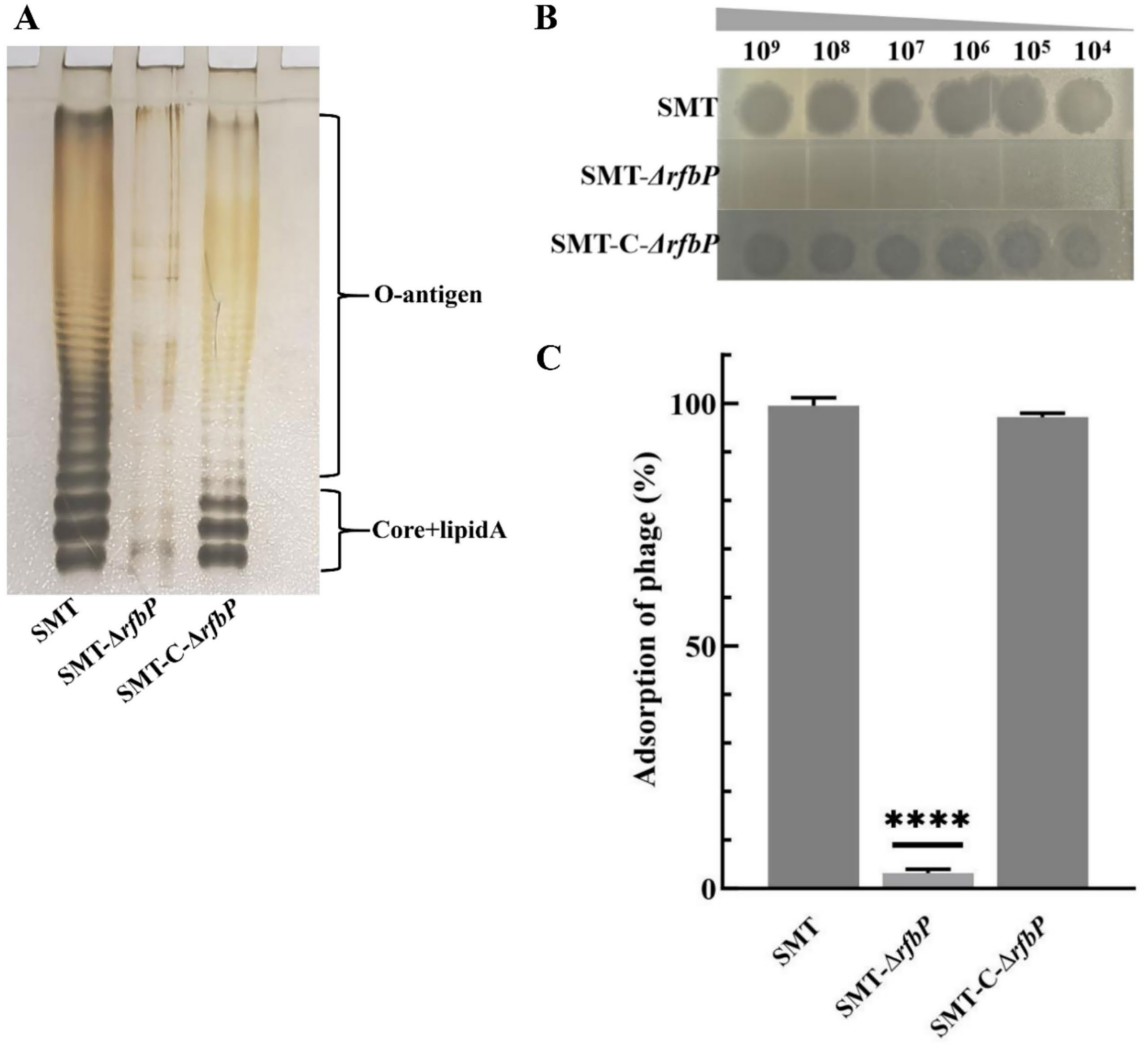
Effect of O-antigen deficiency on phage JN06 infection. **(A)** SDS-PAGE analysis of LPS extracted from wild-type (SMT), SMT-Δ*rfbP*, and complemented (SMT-C-Δ*rfbP*) strains, visualized by silver staining. Extracted LPS samples were loaded onto a 12% polyacrylamide gel and separated by using electrophoresis at 50 V for 20 min followed by 100 V for 90 min. The gel was washed twice with de-ionized water and visualized according to the recommended protocol of the PAGE gel silver staining kit (Solarbio). **(B)** Efficiency of plating (EOP) of phage JN06 on different strains. **(C)** Adsorption efficiency of JN06 to strains SMT, SMT-Δ*rfbP*, and SMT-C-Δ*rfbP*. Data were presented as mean ± SD; ****p < 0.0001.

To further examine the role of O-antigen in phage adsorption, a *rfbP* deletion mutant (SMT-Δ*rfbP*) was constructed. SDS-PAGE analysis of LPS confirmed the absence of O-antigen in SMT-Δ*rfbP*, while the complemented strain (SMT-C-Δ*rfbP*) restored the O-antigen profile (Figure 7A). Phage infectivity assays showed that JN06 was unable to form plaques on the O-antigen-deficient strain SMT-Δ*rfbP* (Figure 7B). Consistently, adsorption assays demonstrated that the adsorption rate of JN06 to SMT-Δ*rfbP* was markedly reduced (3.23%). Restoration of *rfbP* in the complemented strain (SMT-C-Δ*rfbP*) significantly recovered phage adsorption to levels comparable to the wild-type strain (Figure 7C). These results demonstrated that O-antigen is required for efficient adsorption of phage JN06.

### 3.12 Interaction between tail proteins and O-antigen

**Figure 8:**
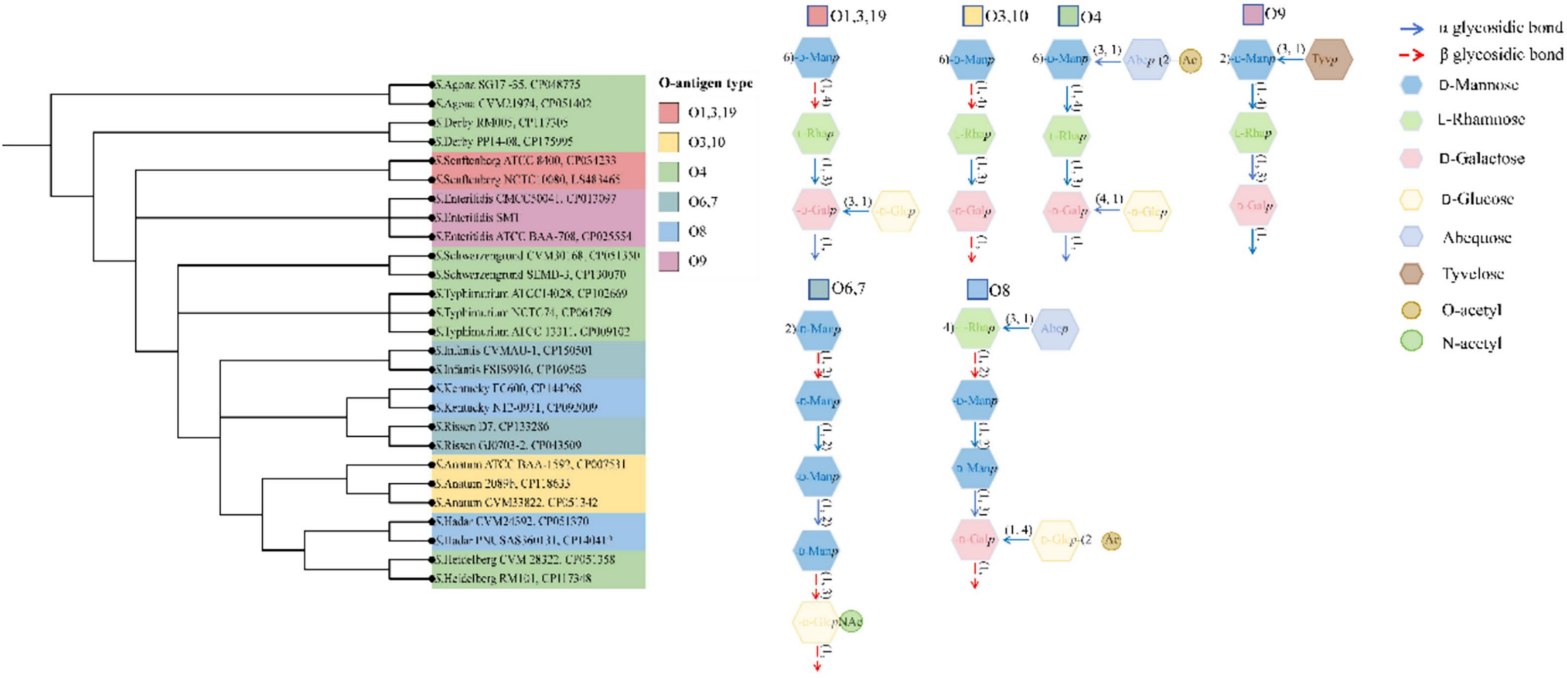
Structural comparison of O-antigen architectures among different *Salmonella* serotypes. The complete genomes of *Salmonella* strains were retrieved from the public database NCBI (www.ncbi.nlm.nih.gov). A phylogenetic tree was constructed by using CVTree (tlife.fudan.edu.cn/cvtree3). D/L: Conformation of monosaccharide.

JN06 exhibited lytic activity against multiple serotypes of *Salmonella* with different drug-resistance profiles, including O1,3,19; O3,10; O4; and O9. These serotypes shared a common architectural feature in their O-antigen backbone, consisting of the trisaccharide repeating unit D-Man*p*-(1 → 4)-L-Rha*p*-(α1 → 3)-D-Gal*p* (the full forms of the abbreviations were shown in Figure 8). We retrieved *Salmonella* genomic data from NCBI and analyzed their serotypes and drug-resistance profiles, found no correlation between serotype and resistance profile (Table 2). JN06’s lytic activity appeared unrelated to bacterial drug resistance profiles. Therefore, we hypothesized that JN06’s lytic activity may be linked to the O-antigen structure rather than bacterial drug-resistance profiles.

**Table 2:** O-antigen and drug-resistance profiles in multiple serotypes of *Salmonella.*

O-antigen	Serotype	Strain name	Origins	Drug-resistant profiles
O1,3,10	Senftenberg	ATCC 8400	ATCC	TOB/AMK
		NCTC 10080	NCTC	TOB/AMK
O3,10	Anatum	2089b	Duck	AMP/AMC/GEN/NA/SXT/TOB/AMK/CIP/CEFEP/TZP/TMP/CML
		CVM 33822	Swine	AMK/AMP/AMC/CTX/CFX/CAZ/TZP/SXT/TCY
		ATCC BAA-1592	ATCC	TOB/AMK
O4	Agnoa	CVM 21974	Bovine	GEN/TOB/AMK/AMP/SXT/TCY/CML
		SG17-135	Silver gull	CIP/AMP/CEFEP/CTX/CAZ/SXT/TMP/AZM
	Derby	PP14-08	Pork	TOB/AMK/CIP/AMP/CEFEP/CTX/CAZ/SXT/TCY
		RM005	*Bos taurus*	TOB/AMK
	Heidelberg	CVM 28322	Swine	GEN/TOB/AMP/AMC/CTX/CFX/CAZ/TZP/SXT/TMP/TCY
		RM101	*Bos taurus*	AMP/AMC/CTX/CFX/CAZ/TZP/SXT/TMP
	Schwarzengrund	SEMD-3	Stool	TOB/AMK
		CVM 30168	Turkey	GEN/ TOB/AMK/AMP/SXT/TCY
	Typhimurium	ATCC 14028	ATCC	TOB/AMK
		ATCC 13311	ATCC	TOB/AMK
		NCTC 74	NCTC	TOB/AMK
O6,7	Infantis	CVMAU-1	Canine	TOB/AMK
		FSIS9916	Chicken breast	TOB/AMK/CIP/NA/SXT/TCY
	Rissen	GJ0703-2	Stool	TOB/AMK/AMP/SXT/TMP/TCY
		D7	Swine liver	TOB/AMK/AMP/SXT/TMP/AZM/TCY
O8	Hadar	CVM 24392	Turkey	TOB/AMK/SXT/TMP/TCY
		PNUSAS360131	-	TOB/AMK
	Kentucky	FC600	Faecel	TOB/AMK/CIP/NA/TCY
		N12-0931	Inguinal swab	GEN/TOB/AMK/CIP/NA/AMP/AMC/CEFEP/CTX/CAZ/IPM/MEMTZP/SXT/AZM
O9	Enteritidis	ATCC BAA-708	ATCC	TOB/AMK
		CMCC 50041	CMCC	TOB/AMK
		SMT	Chicken	GEN/TOB/AMK/CIP/NA/AMP

Genomic information of Salmonella was obtained from the National Center for Biotechnology Information (www.ncbi.nlm.nih.gov). The O antigen and drug-resistance spectrum of Salmonella were predicted using Seqsero (cge.food.dtu.dk/services/SeqSero/) and ResFinder (genepi.food.dtu.dk/resfinder), respectively.

ATCC, American Type Culture Collection; NCTC, National Collection of Type Cultures; CMCC, National Center for Medical Culture Collection.

AMC, amoxicillin; AMP, ampicillin; AMK, amikacin; AZM, azithromycin; CAZ, ceftazidime; CEFEP, cefepime; CFX, cefoxitin; CIP, ciprofloxacin; CML, chloramphenicol; CTX, cefotaxime; FFC, florfenicol; GEN, gentamicin; IPM, imipenem; MEM, meropenem; NA, nalidixic acid; NEO, neomycin; SXT, sulfamethoxazole; TCY, tetracycline; TOB, tobramycin; TMP, trimethoprim; TZP, tazobactam/piperacillin.

**Figure 9:**
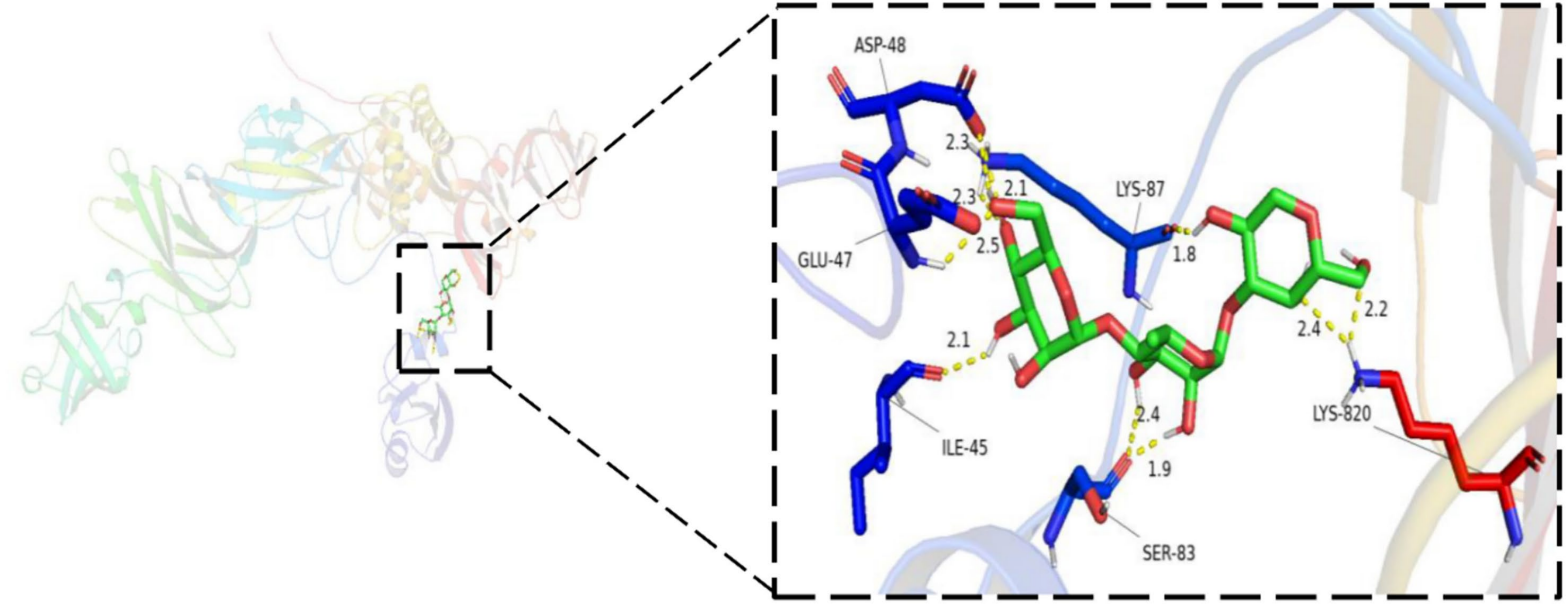
Molecular docking analysis of tail fiber protein with the O-antigen backbone. Predicted binding interaction between tail fiber protein and the trisaccharide motif D-Man*p*-(1 → 4)-L-Rha*p*-(α1 → 3)-D-Gal*p*. Binding energy was calculated as −4.23 kcal/mol. Hydrogen bonds are indicated by yellow dashed lines, and the number above the dashed line indicated the length of the hydrogen bonds.

Molecular docking analysis was performed to evaluate the interaction between phage tail proteins and this conserved O-antigen structure. Among the tested proteins, the tail fiber protein showed the lowest binding free energy (−4.23 kcal/mol), indicating the strongest predicted interaction (Supplementary Table S3). Detailed analysis revealed that the tail fiber protein formed multiple hydrogen bonds with the O-antigen backbone (D-Man*p*-(1 → 4)-L-Rha*p*-(α1 → 3)-D-Gal*p*) through residues ILE-45 (2.1 Å), GLU-47 (2.5 Å), ASP-48 (2.1 Å, 2.3 Å), SER-83 (1.9 Å, 2.4 Å), LYS-87 (1.8 Å) and LYS-820 (2.2 Å, 2.4 Å) (Figure 9). In contrast, docking with the O8 serotype O-antigen resulted in a positive binding energy (2.56 kcal/mol), suggesting weak or unfavorable interaction (Supplementary Table S3). These results indicated that tail fiber protein preferentially interacts with specific O-antigen structures.

## 4 Discussion

In this study, we successfully isolated a lytic and broad-spectrum phage JN06 using MDR *S. enteritidis* as a host. JN06 can effectively lyse various serotypes of *Salmonella*, including typical foodborne pathogenic *Salmonella* serotypes such as *S. enteritidis*, *S.* Typhimurium, *S.* Agona, *S.* Derby, *S.* Anatum, *S.* Schwarzengrund, *S.* Heidelberg and *S.* Senftenberg, which are most frequently identified in vegetables, dairy products, meat, grains, and other foods (Liu et al., 2023; Mansour et al., 2020; Siddique et al., 2022). The broad host range of phages confers them substantial advantages in the control of mixed-pathogen infections (Rodríguez-Rubio et al., 2013). In addition, JN06 displayed a short latent period of 6 min, which is shorter than that reported for several previously described *Salmonella* phages, suggesting rapid amplification after host adsorption (Chahar et al., 2025; Naveen Kumar et al., 2024). These results indicated that JN06 has a wider range of applications and higher lysis performance in pathogen elimination.

Notably, JN06 exhibited remarkable stability under extreme conditions, retaining approximately 50% of its activity at pH 2 and maintaining infectivity at temperatures up to 70 °C. Compared with previously reported *Salmonella* phages, such tolerance is relatively uncommon. Most phages are stable within a narrower pH range (typically pH 4–12) and show reduced stability at temperatures above 60 °C (Song et al., 2024). In some reports, phages are completely inactivated under strongly acidic conditions such as pH 2 (Park et al., 2012). Nevertheless, similar tolerance to extreme acidity has been reported in certain phages. For example, *Salmonella* phage SAL-PG was shown to retain activity at pH 2 despite partial reduction in titer (Rahaman et al., 2014). In addition, some phages have demonstrated stability across a wide temperature range, including up to 70 °C (Juliet et al., 2024). The high stability of phages under different conditions determines their sterilizing effect of phage preparations at the site of infection (Gao et al., 2024). Many environmental factors, such as pH and temperature, can affect the activity of phages (Kimura et al., 2008). The exceptional stability of JN06 highlights its potential as a robust biocontrol agent, particularly in applications involving acidic environments or variable temperature conditions.

The genomic annotation results of phage showed that phage JN06 does not contain any virulence or drug resistance genes, indicating that it is a safe phage. JN06 has the proteins were related to bacterial lysis, including holin, lysozyme, and spanin, which further confirms that JN06 is a lytic phage (Bavda et al., 2021; Liu et al., 2022; Xu, 2021). JN06 showed the highest nucleotide similarity (93.28%) to *Salmonella* phage GSP162, the ANI value remained below the commonly used species-level threshold, supporting that JN06 is genetically distinct from previously reported *Jerseyvirus* phages. Notably, a high degree of conservation was observed in the tail proteins between JN06 and GSP162. As tail proteins function as key receptor-binding proteins (RBPs) responsible for host recognition and adsorption (Yin et al., 2025), this high level of similarity suggests that JN06 and GSP162 may share similar adsorption mechanisms. In contrast, divergence in other genomic regions may account for differences in additional biological properties.

JN06 also showed strong antibiofilm activity against *S. enteritidis* SMT. It significantly inhibited biofilm formation and reduced preformed biofilms, with higher efficacy observed at the higher phage titer. This effect was stronger than that reported for some previously described *Salmonella* phages, such as GSP44 (Gao et al., 2024). Because bacterial cells embedded in biofilms are protected by extracellular matrix and often exhibit reduced susceptibility to antimicrobial agents (Liu et al., 2022). The ability of *Salmonella* to form biofilms is one of the main obstacles for reducing its prevalence in the food supply chain (Paz-Méndez et al., 2017), the antibiofilm activity of JN06 increases its practical relevance for food safety applications.

The lettuce model further demonstrated the antibacterial potential of JN06 in a food matrix. Phage treatment reduced *Salmonella* contamination at both 4 °C and 25 °C, with greater reductions observed under refrigeration. Given that lettuce is commonly consumed raw, effective reduction of *Salmonella* on its surface is of particular importance for food safety (Castro-Ibáñez et al., 2017; Herman et al., 2015). The observed antibacterial activity of JN06 under storage conditions therefore highlights its potential application as a biocontrol agent in fresh produce. However, this model was established under controlled laboratory conditions using artificial contamination and a relatively high phage dose. Further studies under more realistic processing and storage conditions are required to validate the practical applicability of JN06.

A major strength of this study lies in the identification of the adsorption receptor and the proposed structural basis of host recognition. Phenotypic analysis of phage-resistant mutants indicated alterations in O-antigen associated traits, and chemical treatment experiments further showed that phage adsorption was strongly affected by IO_4_^−^ but not by proteinase K, supporting the involvement of a surface polysaccharide receptor. The inhibitory effect of purified LPS on phage adsorption further narrowed the candidate receptor to LPS, the O-antigen is a critical component of LPS (Kalynych et al., 2012). More importantly, construction of an O-antigen-deficient strain via deletion of *rfbP* showed that loss of the O-antigen resulted in a marked reduction in phage infectivity and adsorption capacity, whereas complementation restored phage susceptibility. Together, these findings provide strong functional evidence that the O-antigen is required for efficient adsorption of JN06.

Our results further suggested that JN06 recognizes a conserved structural motif within the O-antigen backbone. The *Salmonella* serotypes susceptible to JN06 share a common trisaccharide backbone, D-Man*p*-(1 → 4)-L-Rha*p*-(α1 → 3)-D-Gal*p*, characteristic of Gal-initiated O-antigen groups. Molecular docking analysis further showed that, among the tested tail-associated proteins, the tail fiber protein exhibited the strongest predicted interaction with this structure, whereas docking with the O8 serotype O-antigen was unfavorable. Although docking results are predictive rather than definitive, when considered together with the mutant and adsorption data, these findings support a model in which tail fiber and O-antigen interactions play a key role in host recognition. This interaction may help explain the broad lytic spectrum of JN06. Notably, the susceptible strains belonged to different serotypes and exhibited diverse antibiotic resistance profiles, yet shared a conserved O-antigen backbone. This observation indicates that the host range of JN06 is more closely associated with O-antigen structure than with antibiotic resistance phenotypes. However, the molecular interaction between the tail fiber protein and the O-antigen was inferred from docking analysis and requires further experimental validation. Future studies should therefore evaluate phage performance across multiple representative strains and food matrices, and further confirm the proposed receptor-binding mechanism at the structural level.

## 5 Conclusion

In this study, we isolated and characterized a broad-host-range lytic phage, JN06, capable of infecting multiple *Salmonella* serotypes, and demonstrated its strong antibacterial activity in biofilm and food models. Importantly, we identified the O-antigen as the adsorption receptor of JN06 and showed that a conserved trisaccharide motif, D-Man*p*-(1 → 4)-L-Rha*p*-(α1 → 3)-D-Gal*p*, likely serves as a key recognition site, providing mechanistic insight into its broad host range. These findings not only highlight the potential application of JN06 in controlling foodborne Salmonella but also advance the understanding of phage-host interactions by linking O-antigen structure to host specificity.

## Data Availability

The complete genome sequence of phage JN06 is available in the NCBI GenBank (https://www.ncbi.nlm.nih.gov/genbank) with accession number PQ411278.

## Footnotes

1
rast.nmpdr.org

2
lowelab.ucsc.edu/tRNAscan-SE

3
genocat.tools/tools/cgview_server

4
cge.food.dtu.dk/services/VirulenceFinder

5
genepi.food.dtu.dk/resfinder

6
glycam.org

7
swissmodel.expasy.org
