# Supplementary material for: Isolation and characterization of a broad-spectrum Salmonella phage targeting featural foodborne serotypes
Source: Front Microbiol. 2026 May 26;17:1827076. doi: 10.3389/fmicb.2026.1827076 (PMC13246685; doi:10.3389/fmicb.2026.1827076)
Supplement: Supplementary file 1 [file Data_Sheet_1.pdf]

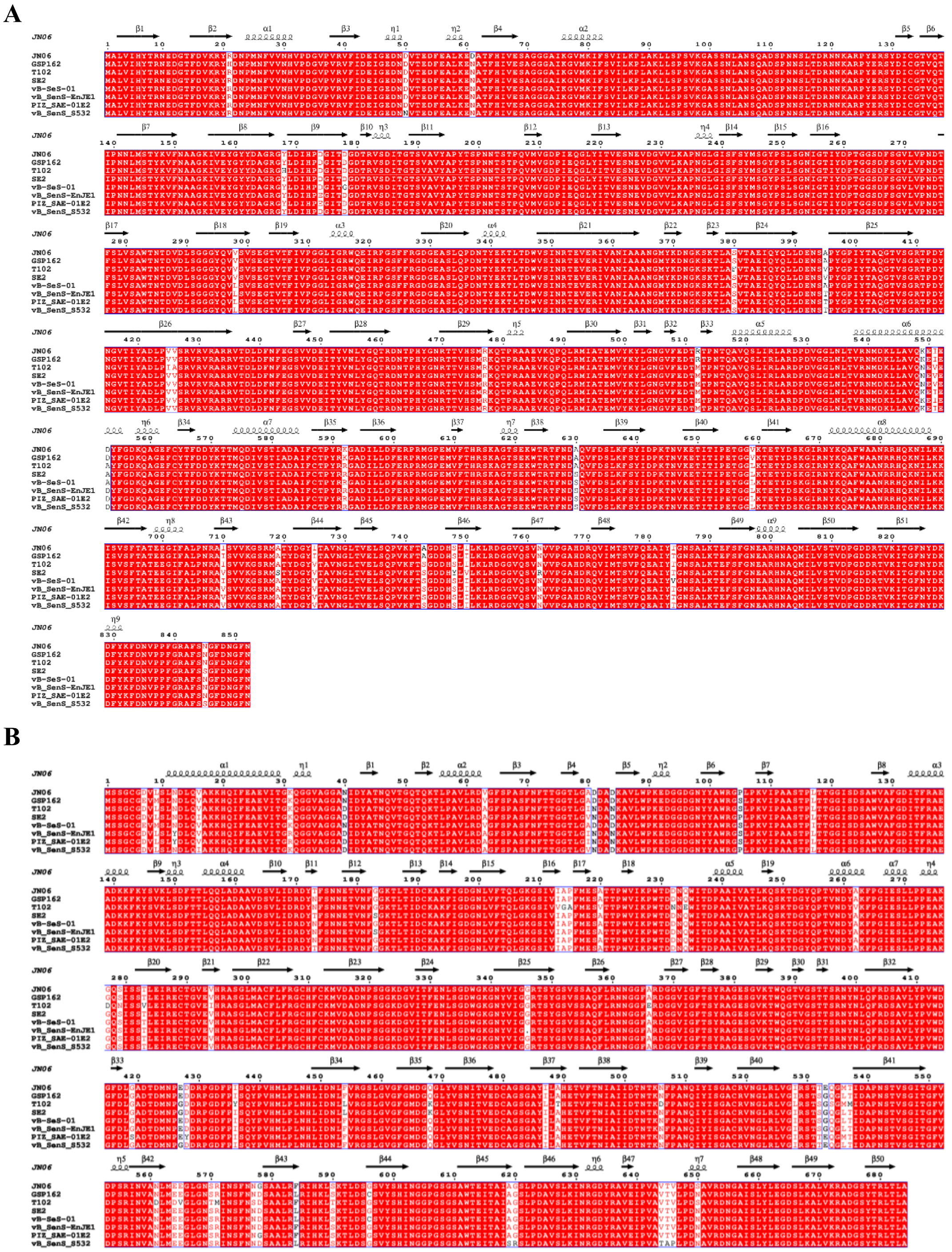

Fig. S1. Multiple alignment of phage tail sequences. (**A**) Tail fiber protein; (**B**) Tail spike protein. Of note, the strain names of phages are indicated on the left.

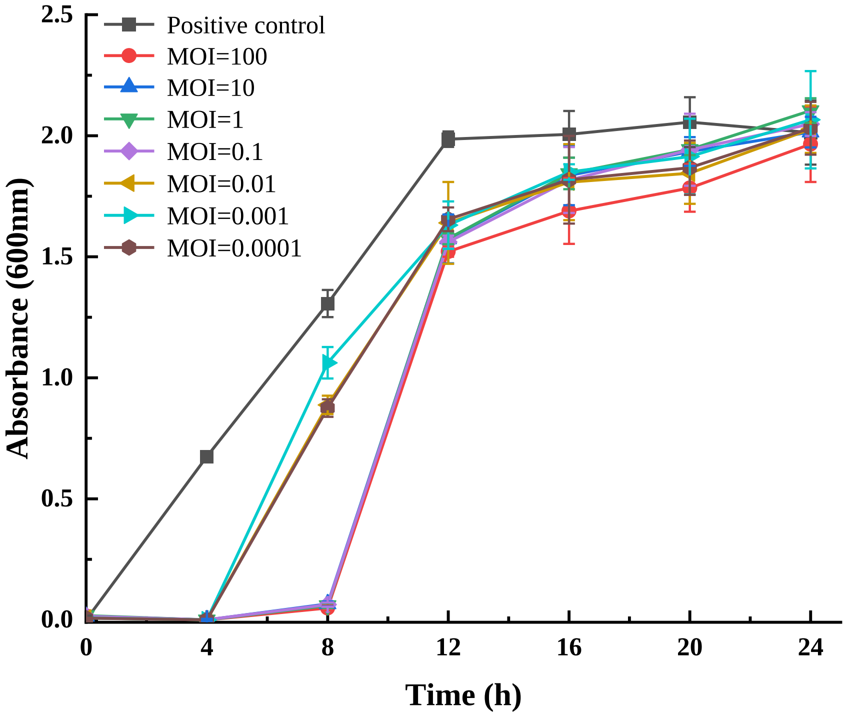

Fig S2. Antibacterial activity of phage JN06 against *S.* Enteritidis SMT *in vitro*.

Antibacterial activity of phage JN06 against *S.* Enteritidis SMT in *vitro* at 37°C. Notably, the values are presented as the average of three replicates, and error bars indicate the standard deviation of independent replicate experiments.

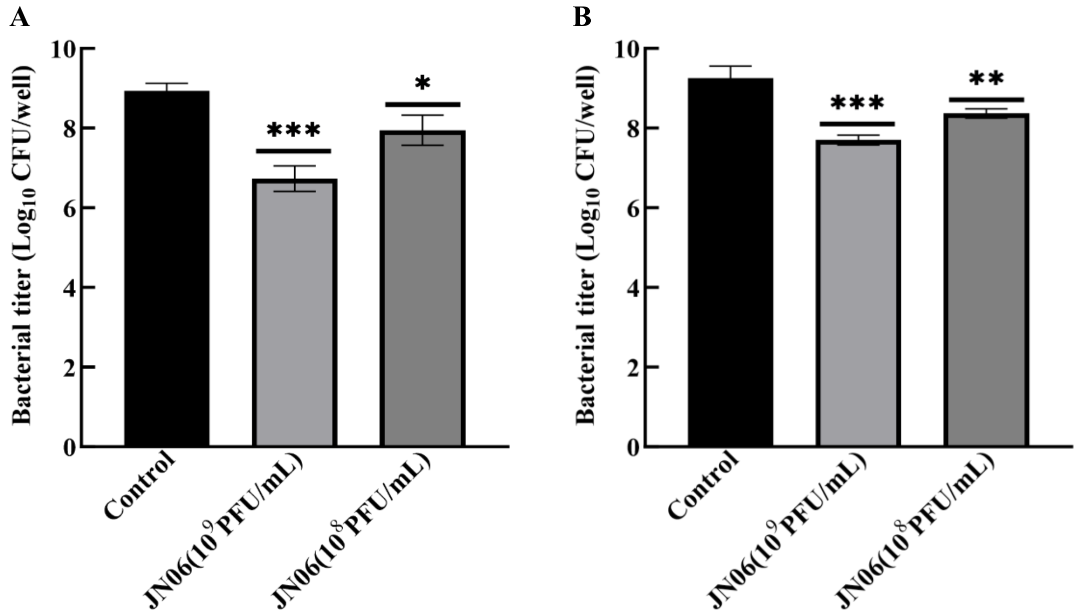

Fig S3. Effect of phage JN06 on viable bacterial counts in biofilms.

(A) Inhibition of biofilm formation. (B) Disruption of pre-formed biofilms. Data are presented as mean ± SD , n=3/group; mean comparisons were performed using one-way ANOVA, followed by Duncan's test. *: p<0.05, **: p<0.01, ***: p<0.001.

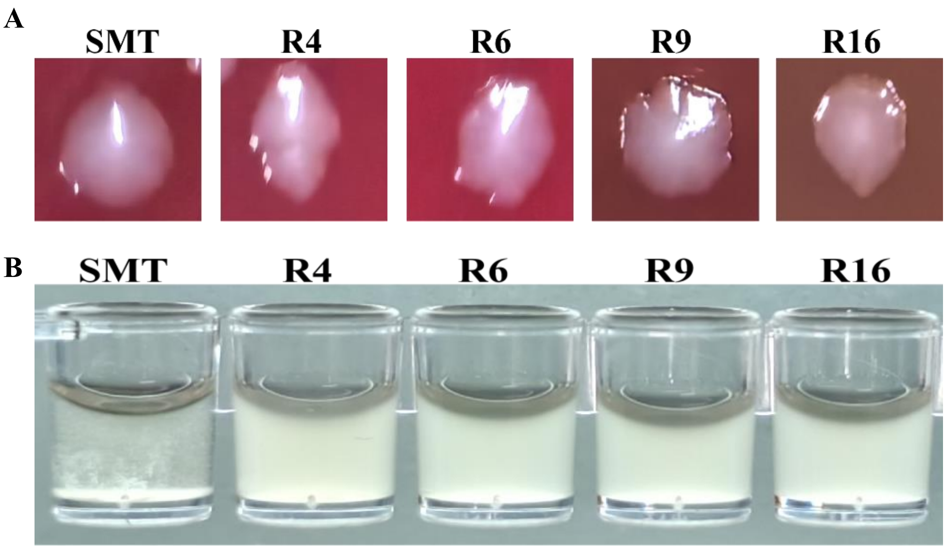

Fig S4. Characterization of the bacteriophage-resistant SMT derivativities. (**A**) The morphological characteristics of the wild-type and the phage-resistant strains on the Columbia blood agar plates. (**B**) Agglutination difference between wild-type and phage-resistant strains.
