# Supplementary material for: Isolation and characterization of a broad-spectrum Salmonella phage targeting featural foodborne serotypes
Source: Front Microbiol. 2026 May 26;17:1827076. doi: 10.3389/fmicb.2026.1827076 (PMC13246685; doi:10.3389/fmicb.2026.1827076)
Supplement: Supplementary file 2 [file Table_1.docx]

**Table S1. Bacterial strains and plasmids used in this study**

| **Strains/plasmids** | **Description** | **Note** |
| --- | --- | --- |
| **Strains** |  |  |
| *S.* Enteritidis SMT | Wild-type strain originating from chicken intestines | Lab stock |
| SMT-Δ*rfbP* | *S.* Enteritidis SMT Δ*rfbP* (gene deletion) | This work |
| SMT-C-Δ*rfbP* | *S.* Enteritidis SMT Δ*rfbP* containing pBAD33-carrying *rfbP* (gene complementation) | This work |
| SMT-R4 | The bacteriophage-resistant SMT derivativities screened by *in vitro* co-culture | This work |
| SMT-R6 |  | This work |
| SMT-R9 |  | This work |
| SMT-R16 |  | This work |
| **Plasmids** |  |  |
| pKD46 | a knockout vector containing the recombinase, Tet^R^ | Lab stock |
| pKD3 | a knockout vector containing homologous arms, Cml^R^ | Lab stock |
| pBAD33 | an arabinose-induced expression vector, Cml^R^ | Lab stock |
| pBAD33::C*rfbP* | Recombinant pBAD33 carrying *rfbP* to complement  SMT-Δ*rfbP* mutant, Cml^R^ | This work |

**Table S2. Primers used for establishing the *rfbP* mutant and complementation strain**

| **Primer** | **Sequence (5′-3′)** |
| --- | --- |
| **Knockout** | |
| rfbp-HF | TTTACTTAATATGCCTATTTTATTTACATTATGCACGGTCAGAGGGTGAGGATTAAATATGAATATCCTCCTTAG |
| rfbp-HR | TTCAGATTTTACGCAGGCTATTTTATACAATTATTATTCAGTACTTCTCGGTAAGCAGCGATTGTGTAGGCTGGAG |
| **Complementation** | |
| P-HB-F | TGCTCTAGAATGGATAATATTGATAATAAGTATAATC |
| P-HB-R | CCCAAGCTTTTAATACGCACCATCTCG |
| **Verification** | |
| IDF | TTATGCACGGTCAGAGGGTG |
| IDR | TGCCGACATGGAAGCCATCA |

**Table S3. Docking parameters of the tail fiber protein and O-antigen backbone assessed by AutoDock Vina**

| **Tail protein** | **Ligand** | **Binding energy [Kcal/mol]** |
| --- | --- | --- |
| Tail fiber protein | D-Man*p*-(1→4)-L-Rha*p*-(α1→3)-D-Gal*p* | -4.23 |
| Tail spike protein |  | -0.78 |
| Minor tail protein |  | -1.65 |
| Tail protein |  | -2.64 |
| Tail fiber protein | L-Rhap-(β1→2)-D-Manp-(α1→2)-D-Manp-(α1→3)-D-Galp | 2.56 |
